# Effect of fractionated therapeutic radiotherapy on surface microhardness and roughness of giomer- and ormocer-based restorative composites: an in vitro study

**DOI:** 10.1038/s41598-026-64330-0

**Published:** 2026-08-05

**Authors:** Hamis Rashwan, Rasha Saad, Nada Sholkamy, Mona Riad

**Affiliations:** 1 Faculty of Dentistry, Minia University, Minia, Egypt; 2 Faculty of Dentistry, Minia University, Minia, Egypt; 3 Faculty of Medicine, Minia University, Minia, Egypt; 4 Faculty of Dentistry, Cairo University, 11 El-Saraya St, Manial, Cairo, 11553 Egypt

**Keywords:** Bioactive restorative material, Microhardness, Organically modified ceramic, Radiotherapy, Surface roughness, Cancer, Health care, Materials science, Medical research, Oncology

## Abstract

The influence of therapeutic radiation on the surface properties of contemporary restorative materials remains an important area of investigation. This study aimed to assess the effect of therapeutic radiation on the microhardness and surface roughness of two restorative materials, as these properties are associated with restoration durability. Ninety-six discs were fabricated from Giomer and Ormocer resin composites (n = 48 per material). Specimens were randomly allocated to two subgroups: surface microhardness (*MH*) and roughness (*Ra*), based on the tests performed (n = 24). For each test, specimens were further subdivided into two subgroups (n = 12). IR0: control, no irradiation; IR1: irradiated, exposed to a total of 70 Gy over 35 days (2 Gy/day, 5 days/week) using a linear accelerator (10 MV photons, 2 Gy/fraction). *MH* was measured using a 100 g load for 15 s, and *Ra* was evaluated using a non-contact digital microscopy method and analyzed with WSxM software. Data were analyzed using two-way ANOVA, with significance set at *p* < 0.05. No significant differences were detected between control and irradiated specimens in either microhardness or surface roughness (*p* > 0.05). Both restorative materials demonstrated stable surface microhardness and roughness, following exposure to a clinically relevant radiotherapy protocol.

**Supplementary Information:**

The online version contains supplementary material available at 10.1038/s41598-026-64330-0.

## Introduction

Radiotherapy is a primary treatment approach for malignant tumors of the head and neck region and is typically delivered using high-energy rays at cumulative doses of 40–70 Gray^1^. Although associated with therapeutic benefits, radiotherapy can have complex oral side effects that involve the oral mucosa, bone, dentition, and salivary glands, such as hyposalivation, mucositis, loss of taste, trismus, radiation caries, and osteoradionecrosis^2^. With the absence of salivary lubrication and the accompanying absence of the cleansing effect, staining, biofilm buildup, and chemical degradation of the restorative surfaces are more likely to occur with these subtle radiation-induced surface degradation and to intensify the clinical impacts of these surface changes^3–5^.

Direct restorative materials, used to restore carious lesions in irradiated patients, commonly include resin composites (RCs) and glass ionomer-based materials, which can have reduced clinical performance in post-radiotherapy environments^6^. The presence of ionizing radiation in the resin matrix can lead to the formation of reactive species and free radicals^7,8^, which can cause further cross-linking reaction, post-curing reaction, chain scission, or degradation of the polymer network^9^. These physicochemical mechanisms may influence surface-related properties such as microhardness and roughness, with the magnitude and direction of the effect depending on material composition, filler technology, and irradiation protocol^7,8,10,11^. Consequently, concerns have been raised regarding the long-term clinical performance and durability of restorations placed in patients receiving head and neck radiotherapy, as reduced restoration longevity may necessitate repeated dental interventions^12^, thereby increasing the burden of oral health management in these patients^11^.

In clinical service, restorative materials undergo time-dependent degradation due to mechanical, chemical, and thermal stresses^13,14^. In irradiated patients, these degradation processes may be further accelerated by the aggressive oral environment associated with hyposalivation, altered saliva composition, and shifts in oral microflora^2^. Even minor radiation-induced alterations may adversely influence restoration longevity in the oral environment associated with head and neck radiotherapy^10^.

Surface microhardness (MH) and roughness are sensitive parameters used to assess the effects of radiation on the polymer structure and the integrity of the filler-matrix interface^6^. Surface microhardness is commonly used to assess a material’s resistance to wear. Changes in microhardness may reflect alterations in cross-link density and surface degradation, which may influence restoration longevity^15^. Likewise, surface roughness may be affected by radiation-related matrix degradation or filler debonding. Alterations in surface roughness are clinically relevant, as increased roughness facilitates bacterial adhesion and staining, thereby raising the likelihood of secondary caries and esthetic deterioration^16,17^.

In response to the enhanced demineralization risk associated with irradiated oral environments, increasing attention has been directed toward resin-based restorative materials that incorporate bioactive and ion-releasing components, capable of releasing remineralizing ions over extended periods, which may reduce mineral loss and improve resistance to acid challenges at the tooth-restoration interface^6,18,19^. Because ionizing radiation primarily interacts with organic polymer network^8,20^, restorative materials with different matrix chemistry and filler characteristics may respond differently to therapeutic radiation^21–24^. Restorative materials capable of maintaining structural integrity and surface stability under these challenging oral conditions may offer potential clinical advantages. A low-shrinkage nano-hybrid Giomer composite was selected as one of the investigated materials.

Giomer-based materials incorporate surface pre-reacted glass-ionomer (S-PRG) fillers within a resin matrix, enabling the sustained release and recharge of multiple bioactive ions, including fluoride, strontium, sodium, silicate, phosphate, and borate^25^. The homogeneous distribution and stable integration of S-PRG fillers within the resin matrix may limit filler debonding and surface degradation under mechanical and chemical stress^18,26^. The unique combination of S-PRG fillers and a low-shrinkage resin matrix may confer greater resistance to radiation-induced degradation than conventional resin composites^22,24,25^.

Ormocer-based composites are inorganic–organic hybrid materials based on an organically modified siloxane network. Unlike conventional dimethacrylate-based composites, ormocers are formulated with pure silicate technology, where both the inorganic fillers and the polymer matrix are based on silicon oxide chemistry, resulting in a fundamentally distinct matrix architecture^27,28^. The highly cross-linked inorganic–organic copolymer structure of ormocers provides enhanced network stability and improved resistance to hydrolytic and chemical degradation under challenging oral conditions^29,30^. Previous investigations have demonstrated favorable degradation resistance and clinical performance of Ormocer-based restorative systems, supporting their use as contemporary low-shrinkage restorative materials^31–33^. Given the fundamental differences in matrix chemistry, polymer architecture, and filler technology between these two material categories and their structural distinction from conventional dimethacrylate-based composite, they represent two contemporary restorative concepts with fundamentally different filler technologies and matrix architectures. Since radiation-induced effects are expected to be material-dependent, comparing these systems may provide insight into how distinct restorative technologies respond to clinically simulated fractionated therapeutic ionizing radiation.

Because radiation-induced effects on resin-based restorative materials are believed to depend on material composition, matrix architecture, and filler technology^22,24,34^. This in vitro study was designed to evaluate the effect of fractionated therapeutic ionizing radiation on the surface microhardness and surface roughness of a Giomer-based and an ORMOCER-based restorative material. The null hypotheses tested were:

H01: Fractionated therapeutic ionizing radiation of 70 Gy would not significantly affect the surface microhardness of Giomer and Ormocer.

H02: Fractionated therapeutic ionizing radiation of 70 Gy would not significantly affect the surface roughness (Ra) of Giomer and Ormocer.

## Materials and methods

### Study design

This laboratory study utilized a two-factor factorial experimental design. The independent variables were radiation exposure (with or without ionizing radiation) and restorative material type (Giomer-based and Ormocer-based restorative composites). Surface microhardness and roughness were the main outcome measures. Non-irradiated specimens served as controls.

### Sample size calculation

The required sample size was determined utilizing statistical PASS software (PASS version 11; NCSS LLC, Kaysville, UT, USA)^35^, with a statistical power of 80% (β = 0.20) and a significance level of α = 0.05 (95% confidence level). Based on a previous study by de Amorim et al.^11^, an effect size (Cohen’s d) of 1.37 was calculated. Calculations indicated that at least 12 specimens per group were required to detect statistically significant differences in the outcome variables between groups.

### Sample preparation

A total of ninety-six disc-shaped specimens were prepared, with 48 specimens fabricated from each restorative material: Giomer-based resin composite (Beautifil II LS; Shofu, Kyoto, Japan) and Ormocer-based resin composite (Admira Fusion; VOCO GmbH, Cuxhaven, Germany). The tested materials were selected as representative examples of Giomer- and ORMOCER-based restorative systems; their compositions and manufacturer details are summarized in Table 1. Custom-made Teflon split molds with a single cylindrical cavity (2 ± 0.1 mm thickness and 10 ± 1 mm diameter) were used^11^. The mold was positioned on a polyethylene terephthalate (PET/Mylar) strip over a glass slab, and the mold was filled with RCs using a gold-plated composite applicator. The upper surface was covered with a Mylar strip and a glass slab, and a standardized load of 20 N (≈ 2 kg) was applied to extrude excess material, create flat specimen surfaces, and minimize the oxygen-inhibited layer^36^.

**Table 1:** Materials used, their specifications, composition, manufacturer, and batch number.

Restorative materials	Specification	Composition		Filler content (wt%)	Manufacturer	Batch No
		Matrix	Filler			
Beautifil II LS	Giomer	Bis-GMA, TEGDMA and UDMA	Multifunctional aluminofluoro-borosilicate glass filler, aluminum oxide, S-PRG silica,prereacted glass ionomer filler, camphoroquinone	wt% 68–83	Shofu, Kyoto, Japan	012,377
Admira Fusion	Ormocer	Inorganic–organic hybrid Ormocer matrix with organically modified siloxane network	glass ceramics (1 µm), Silicon oxide Nano filler, Pigments	84% (wt%)	Voco GmbH, Cuxhaven, Germany	2,146,606

Bis-GMA, Bisphenol A diglycidylmethacrylate; UDMA, Urethane dimethacrylate; TEGDMA, Triethyleneglycol dimethacrylate.

A fully charged LED light-curing device (Radii Plus; SDI, Bayswater, Victoria, Australia) with an output intensity of 1500 mW/cm^2^ was used for 20 s, according to the manufacturer’s instructions, in direct contact with the restoration^37,38^. Periodic verification of the light intensity was performed using an integrated radiometer (Bluephase Meter II; Ivoclar Vivadent, Schaan, Liechtenstein). After removal from the molds, specimen dimensions were verified using a digital caliper.

All specimens were stored in distilled water at 37 °C. Finishing and polishing were then performed by a single operator to minimize inter-operator variability, using a Sof-Lex disc system (3 M ESPE; St. Paul, MN, USA) in a sequential manner (coarse, medium, fine, and superfine) for 20 s per disc, using unidirectional strokes under standardized light pressure and continuous water cooling. Specimens were thoroughly rinsed and ultrasonically cleaned after polishing.

All specimens were visually inspected under × 10 magnification using a stereomicroscope to verify surface integrity and dimensional accuracy. Specimens exhibiting defects like voids or surface irregularities were discarded before testing.

Following specimen inspection, all 96 composite resin discs were numerically labelled and randomly allocated to two subgroups. Randomization was performed using the RAND function in Microsoft Excel (Microsoft, Redmond, WA, USA). Outcome assessment measurements were performed by an examiner blinded to group allocation, and statistical analysis was conducted on coded data with group identity revealed only after completion of analysis.

### Specimen grouping and experimental design

Specimens were grouped based on the type of test, restorative material, and radiation exposure, as shown in Table 2. In every test, the two restorative materials were directly evaluated under both non-irradiated and irradiated conditions.

**Table 2:** Experimental design.

Test	Material	Control (non-irradiated)	Irradiated	Total
Microhardness	Beautifil II LS	12	12	24
	Admira Fusion	12	12	24
Surface roughness	Beautifil II LS	12	12	24
	Admira Fusion	12	12	24
Total		48	48	96

### Irradiation protocol

A board-certified radiation oncologist and a medical physicist supervised the irradiation procedures at Minia Oncology Center, Egypt, in order to ensure the accuracy and safety of the treatments. The specimens were positioned in holes cut in specifically designed Styrofoam molds so that all specimens would align uniformly. The samples were completely immersed in distilled water during irradiation to ensure even dose distribution (Supplementary Fig. S1). This modality replicates the clinical application of thermoplastic immobilization masks during head-and-neck radiotherapy (Supplementary Fig. S2), which maintain patient positioning without altering the radiation dose administered^39^. The samples were sandwiched between two solid water-equivalent phantoms (15 cm height; BTW, Germany), positioned above and below the samples to create homogeneous lateral and backscatter radiation. A bolus material was applied over the surface to ensure dose buildup, and all specimens were aligned so that the isocenter matched the geometric center of the sample container. A computed tomography (CT) simulation was performed (Supplementary Fig. S3), and the images were transferred to the treatment planning software (Monaco; Elekta, Stockholm, Sweden)^40^, where a three-dimensional radiation plan was generated using two opposing photon beams (anterior and posterior) with 10 MV photon energy (Supplementary Fig. S4). Treatment was administered using the medical linear accelerator (Elekta Synergy; Elekta, Stockholm, Sweden)^41^, with precise dose coverage and no cold or hot spots. The prescribed dose was 70 Gy, delivered in 2 Gy fractions, five fractions per week, over 7 weeks^1,42^. To preserve specimens’ hydration, all specimens were kept fully submerged in distilled water at 37 °C, with the water replaced daily.

### Testing procedures

#### Microhardness test

A Digital Display Vickers Micro-hardness Tester (HVS-50; Laizhou Huayin Testing Instrument, Laizhou, China) with a Vickers diamond indenter and a 20 × objective lens was used to measure the surface micro-hardness of each specimen. A load of 100 g was applied to the surface for 15 s. For each specimen, three indentations were made, arranged evenly around a circle and kept at least 0.5 mm apart. The diagonal lengths of the indentations were measured using a built-in scaled microscope, and Vickers values were converted into corresponding micro-hardness values. The mean value of the three indentations was determined and utilized for statistical analysis.

### Microhardness calculation

Micro-hardness was calculated from the equation below:

$${\mathrm{HV}} = {1}.{\text{854 P}}/{\mathrm{d}}^{{2}} ,$$

where HV is the Vickers hardness number (kgf/mm^2^), P is the applied load (kgf), and d is the mean diagonal length of the indentation (mm).

### Surface roughness test

A non-contact optical method was employed to evaluate surface roughness, which allows the surface topography to be quantified without physical interference^43^. Surface images were acquired utilizing a digital microscope (U500 × Digital Microscope; Guangdong, China) equipped with a 3-megapixel digital camera, positioned vertically at a fixed working distance of 2.5 cm from the specimen surface. Illumination was provided by eight adjustable LED light sources with a color rendering index of approximately 95%, arranged at an angle of nearly 90° relative to the optical axis.

**Fig. 1:**
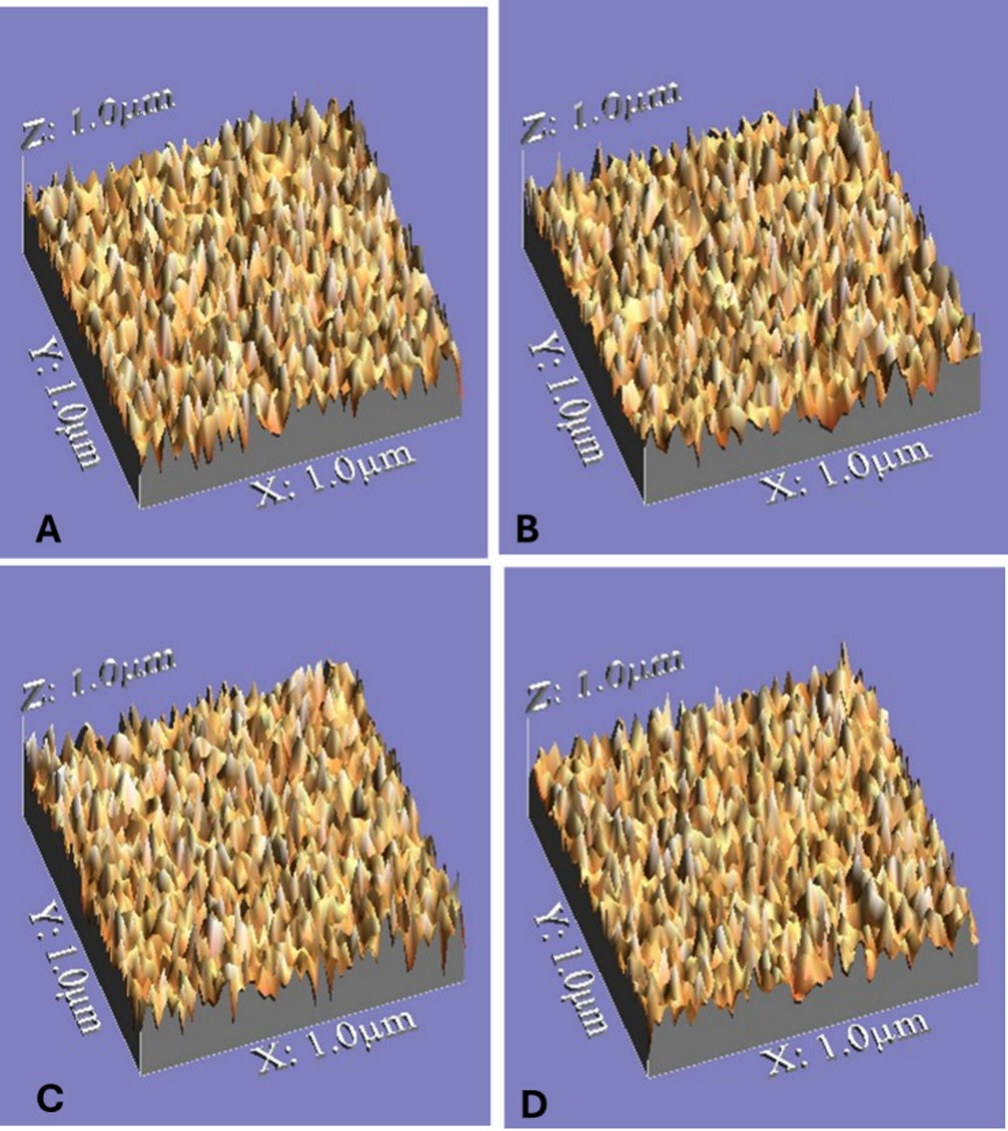
Three-dimensional surface topography images of the tested materials. (**A**) Ormocer before irradiation, (**B**) Ormocer after irradiation, (**C**) Giomer before irradiation, and (**D**) Giomer after irradiation. The surface morphology appeared comparable before and after irradiation.

Images were captured at a fixed magnification of 90 × and maximum resolution (1280 × 1024 pixels) and transferred to a personal computer for analysis (Fig. 1). To standardize the evaluation area, the digital microscope images were cropped to a specific region of interest (350 × 400 pixels), and calibration was performed using surface analysis software (WSxM; Nanotec Electronica, Madrid, Spain) to convert pixel dimensions into physical units^44^. Calibration was performed by finding a reference object of known dimensions on the software scale to convert pixel values to real-world units. Following calibration, standardized surface roughness measurements were performed within predefined analysis windows.

Surface roughness parameters (Ra) were subsequently evaluated within calibrated analysis areas of 10 µm × 10 µm. The WSxM software was used to determine the maximum surface height and average surface roughness (Ra, µm), which are widely accepted parameters for surface roughness evaluation. Each sample was measured three times at different positions within a 5 mm radius of the center. After that, the Ra readings were averaged, and this average served as the statistical value considered in the analysis^45^.

### Statistical analysis

Numerical data were presented as mean ± standard deviation (SD) values. They were explored for normality and variance homogeneity by checking the data distribution and using Shapiro–Wilk and Levene tests, respectively. Data were normally distributed with homogeneous variances. Microhardness and surface roughness data were analyzed using two-way factorial ANOVA. Simple effects comparisons were made utilizing the ANOVA error term, with p-values adjusted using the False Discovery Rate (FDR) method. The significance level was set at *p* < 0.05. Statistical analysis was performed with R statistical analysis software version 4.5.1 for Windows^46^.

## Results

### Microhardness

#### Effect of variables and their interaction

Testing the variables individually and their interaction revealed that neither the restorative material, irradiation, nor their interaction showed any significant difference detected (*p* > 0.05) (Table 3).

**Table 3:** Effect of different variables and their interactions on Microhardness (*MH*).

Source	Sum of Squares (Type III)	df	Mean Square	f-value	*p*-value
Restorative material	0.81	1	0.81	**0.23**	**0.640**
Irradiation	0.67	1	0.67	**2.77**	**0.115**
Restorative material * Irradiation	0.05	1	0.05	**0.20**	**0.658**

df, degree of freedom; not statistically significant (*p* > 0.05).

Comparisons and summary statistics of Vickers microhardness for non-irradiated **(IR0)** and irradiated **(IR1)** for both restorative materials are presented in Table 4 and (Supplementary Fig. S6).

**Table 4:** Comparisons and summary statistics of Microhardness (*MH*) for non-irradiated (IR0) and irradiated (IR1) specimens.

Restorative materials	Hardness (Mean ± SD)		*p*-value
	IR0	IR1	
Giomer	77.62 ± 1.49	77.97 ± 1.14	**0.154**
Ormocer	77.39 ± 1.47	77.59 ± 1.40	**0.403**
*p*-value	**0.732**	**0.573**	

ns, not statistically significant (*p* > 0.05).

For the Giomer-based material, the mean microhardness values were 77.62 ± 1.49 for **IR0** and 77.97 ± 1.14 for **IR1**, with no statistically significant difference detected (*p* = 0.154). Similarly, for the Ormocer-based material, the mean values were 77.39 ± 1.47 for **IR0** and 77.59 ± 1.40 for **IR1**, with no significant difference (*p* = 0.403).

Overall, the effect of irradiation on Microhardness (*MH*) was not statistically significant (*p* > 0.05) for either restorative material.

### Surface roughness

Testing each variable individually, as well as their interaction, revealed that neither the restorative material, nor irradiation, nor their interaction had a statistically significant effect on surface roughness (*p* > 0.05) (Table 5).

**Table 5:** Effect of different variables and their interactions on surface roughness.

Source	Sum of Squares (Type III)	df	Mean Square	f-value	*p*-value
Restorative material	1e-03	1	1e-03	**2.07**	**0.170**
Irradiation	1e-04	1	1e-04	**0.25**	**0.621**
Restorative material * Irradiation	5e-04	1	5e-04	**0.95**	**0.344**

df, degree of freedom; not statistically significant (*p* > 0.05).

Comparisons and summary statistics of surface roughness (Ra, µm) for non-irradiated **(IR0)** and irradiated **(IR1)** specimens of both restorative materials are presented in Table 6 and (Supplementary Fig. S7).

**Table 6:** Comparisons and summary statistics of Surface roughness (Ra) for non-irradiated (IR0) and irradiated (IR1).

Restorative materials	Surface roughness Ra (µm) (Mean ± SD)		*p*-value
	ns:	ns:	
Giomer	0.26 ± 0.02	0.27 ± 0.03	**0.301**
Ormocer	0.27 ± 0.02	0.27 ± 0.02	**0.738**

Not statistically significant (*p* > 0.05).

For the Giomer material, the mean **Ra** values were 0.26 ± 0.02 µm for **IR0** and 0.27 ± 0.03 µm for **IR1**, with no statistically significant difference detected (*p* = 0.301). Similarly, for the Ormocer material, the mean Ra values were 0.27 ± 0.02 µm in both the IR0 and IR1 groups, with no statistically significant difference (*p* = 0.738).

Overall, surface roughness values remained comparable following irradiation, indicating that the applied radiotherapy protocol did not induce measurable surface alterations in either restorative material.

## Discussion

Head and neck radiotherapy creates a challenging oral environment characterized by hyposalivation and increased caries susceptibility^2,47^. Due to the aggressive nature of this oral environment and the low tolerance for repeated restoration replacement, restorative materials placed in patients scheduled for radiotherapy should maintain surface properties, mechanical stability, and esthetic performance after radiation exposure^4,13,48,49^.

The impaired oral conditions of irradiated head and neck cancer patients suggest that a restorative material, such as a low-shrinkage nano-hybrid Giomer composite, may be advantageous, as its surface-pre-reacted glass-ionomer (S-PRG) fillers enable bioactive ion release, which may enhance caries resistance^25^. Beautifil II LS was selected for its specific material composition, which may directly affect surface microhardness and roughness behavior under irradiation. A comparatively high filler loading, coupled with the resin matrix, favors cross-link density and indentation resistance^26^, both of which are closely related to surface microhardness. Moreover, the inclusion of surface-pre-reacted glass ionomer (S-PRG) and pre-polymerized fillers can also increase the interfacial stability of the fillers with the matrix, thereby reducing filler debonding or surface irregularities when exposed to radiation. These geometric characteristics render Beautifil II LS a suitable model for studying the effects of radiation on both surface hardness and surface roughness^6,18,19,50^.

Admira Fusion was selected as an Ormocer-based composite, representing an alternative resin matrix technology to conventional composites. Its inorganic–organic hybrid polymer network is designed to reduce conventional methacrylate content, potentially enhancing dimensional stability, reducing water sorption, and improving degradation resistance^31,38^.The highly cross-linked inorganic–organic copolymer structure of ormocers provides enhanced network stability and improved resistance to hydrolytic and chemical degradation under challenging oral conditions^29,30^. The comparison of Giomer-based and Ormocer-based materials is justified because their distinct matrix chemistries and filler technologies, S-PRG fillers in resin matrix versus organically modified siloxane network, may confer differential responses to ionizing radiation.

In this regard, surface microhardness and surface roughness were selected as clinically relevant, radiation-sensitive indicators. Surface microhardness (MH) reflects the restorative material’s resistance to permanent deformation and is commonly used to correlate laboratory findings with intraoral performance, particularly with respect to wear resistance^15^. Exposure to ionizing radiation may theoretically influence MH through two opposing mechanisms: disruption of the polymer network, leading to reduced hardness, or radiation-induced post-curing effects that promote additional cross-linking within the resin matrix. The latter mechanism involves increased mobility of reactive species, allowing residual C = C bonds to participate in further polymerization, thereby enhancing the degree of conversion and cross-link density^34^. Therefore, the present study was conducted to evaluate the effect of therapeutic ionizing radiation on the surface microhardness and surface roughness of two advanced resin-based restorative materials under controlled in vitro conditions.

Similar post-curing effects have been reported following the thermal treatment of RCs. During thermal post-curing, resin composites typically retain a fraction of unreacted methacrylate double bonds (C = C) and low–molecular-weight species entrapped within the polymer network, which enhances molecular mobility within the partially polymerized network. This increased mobility enables residual C = C bonds and trapped reactive species to diffuse and participate in further polymerization reactions; as a result, improvements in mechanical properties such as microhardness are often noted following thermal treatment of resin composites^51^.

Radiation has been reported to induce molecular excitation and the production of free radicals within the polymer matrix^8,9,20^. The energy transfer may increase the mobility of reactive species (ROS) and may promote further cross-linking reactions within the polymer chains^52,53^. Ionizing radiation may act as a secondary activation stimulus that could promote further polymerization of residual double bonds and potentially increase the structural integrity of the polymer network^10,20^. This radiation-induced post-curing effect may help explain the increased microhardness reported for several resin-based materials following irradiation^54^.

Nevertheless, unlike controlled thermal treatment, ionizing radiation can trigger additional competing degradation mechanisms simultaneously, as the entrapment of free radicals within polymer chains can cause chain scission and bond rupture^20^. Thus, the ultimate mechanical performance depends on the equilibrium between radiation-induced cross-linking and polymer degradation, a process influenced by material composition, irradiation protocol, and radiation dose^10,52^. Based on these competing mechanisms, the present findings were interpreted in the light of the proposed null hypotheses.

Both null hypotheses (H01 and H02) were accepted, as therapeutic irradiation did not produce statistically significant changes in either surface microhardness or surface roughness of the tested restorative materials. In the present study, exposure to therapeutic ionizing radiation did not yield statistically significant changes in the surface microhardness of the restorative materials tested. The present findings are consistent with several studies reporting that radiotherapy exerts neither detrimental nor beneficial impacts on the microhardness of RCs and glass hybrid restorative materials^24,34,55,56^. This outcome may be explained by the presence of two opposing radiation-related mechanisms acting within resin composites.

Ionizing radiation may promote additional cross-linking through post-curing effects, while simultaneously inducing chain scission and polymer degradation^8,20^. The balance between these opposing processes may explain the stability observed in the present findings^24^. These findings are consistent with previous studies reporting minimal or no significant changes in the mechanical properties of resin-based materials following therapeutic irradiation^10,52^. A systematic review^15^ further concluded that a deleterious effect of radiotherapy on microhardness is unlikely, as this conclusion was attributed to the composite resin microstructure, which appears highly resistant to the destructive effects of radiotherapy, particularly when clinically relevant radiation doses are applied^57,58^.

Nevertheless, several studies reported improvements in microhardness and attributed their findings to radiation-induced cross-linking within the polymer chains^54,57,59^. Similarly, Behr et al.^60^ reported significant improvements in hardness, fracture toughness, and wear resistance following electron-beam irradiation of dental composites. However, their study employed industrial irradiation doses ranging from 25 to 200 k Gy, which are substantially higher than the therapeutic radiation dose used in the present study (70 Gy). These differences in irradiation modality and absorbed dose may explain the discrepancy between their findings and the absence of significant changes observed in the current investigation.

However, other studies have reported reductions in microhardness following irradiation of glass ionomers and RCs, attributing these effects to radiation-induced polymer degradation including chain scission caused by high-energy exposure^10,14,61^.

In terms of surface roughness, radiation exposure has been suggested to affect the integrity of the resin–filler interface, potentially leading to filler debonding or differential wear between the resin matrix and filler particles^34^. Such effects are particularly relevant in materials with heterogeneous filler size distributions, where the protrusion or loss of filler particles may contribute to surface irregularities even in the absence of measurable changes in bulk mechanical properties^14,17^.

Surface roughness is influenced by the amount, type, and size of filler particles, which play a critical role in determining the surface characteristics of resin composites (RCs) and should be considered when evaluating surface roughness (Ra)^17^. Beyond radiation-induced chemical alterations, surface roughness may also be influenced by changes at the filler–matrix interface and surface topography stability. Ionizing radiation may induce degradation at the resin–filler interface through free radical formation and polymer chain scission, reducing interfacial integrity and increasing the susceptibility to filler debonding and surface irregularities^9,20^. Any disruption of the interfacial bond between the resin matrix and filler particles may facilitate filler loss, particle protrusion, or localized surface defects, thereby increasing surface roughness^14,15,34^.

The absence of significant changes in surface roughness observed in this study may be attributed to the compositional stability of the tested restorative materials. Both Giomer and Ormocer-based materials are designed to maintain surface integrity under challenging oral conditions. The presence of S-PRG fillers in the Giomer-based composite may contribute to surface stability through ion exchange and acid-neutralizing capabilities, while the highly cross-linked inorganic–organic network of the Ormocer-based material limits matrix softening and filler–matrix degradation. Together, these characteristics may have minimized surface topographical alterations following radiation exposure^18,25,31,56^.

Similar findings have been reported in studies demonstrating that surface roughness remains unchanged following therapeutic radiation exposure^10,14,22^. The unchanged surface roughness observed in these studies was attributed to the use of clinically relevant radiation doses and materials with stable filler–matrix interfaces^22,24,56^, which appear to resist radiation-induced surface degradation. This suggests that, under practical radiotherapy protocols, certain contemporary resin-based materials may retain surface smoothness even after irradiation^10,22^.

Other investigations, however, reported increased surface roughness after radiotherapy in composites, glass ionomers, and resin-modified glass ionomers^11,55^. Such findings were explained by radiation-induced excitation and ionization within polymer matrices, which initiate free radicals and further rupture of polymer chains, altering surface properties, and causing filler particles exposure^11,52^.

These material-dependent alterations are also supported by microscopic evidence. According to a scanning electron microscopy analysis by de Amorim et al.^11^, radiation exposure may reveal filler particles in the subsurface layers of RCs and may even strip the resin coating from glass ionomers, leading to surface roughening that varies with the size of the exposed particles. Consistent with this material-dependent behavior, other studies reported that an increase in surface roughness was observed only in conventional glass ionomers, whereas no significant changes in Ra were observed in resin-modified glass ionomers and RCs^15,24,34^.

This investigation directly addressed the stated aims by evaluating the effect of therapeutic radiation at 70 Gy on surface microhardness and roughness of Giomer-based and Ormocer-based restorative materials. Both null hypotheses (H01 and H02) were accepted, indicating that neither surface property of the materials used was significantly altered by therapeutic radiation doses. These findings confirm the surface integrity of both Giomer-based (Beautifil II LS) and Ormocer-based (Admira Fusion) composites under clinically relevant fractionated radiotherapy protocols and thus add to the scarce data on the radiation response of contemporary restorative materials.

The observed effects of the different radiations on the restorative materials depend on material type, matrix composition and radiation protocol^11,14,15,24^, and, therefore, are material specific, highlighting the need for caution in the choice of materials for patients scheduled for head and neck radiotherapy.

The results of this study should be considered in light of some of the strengths of the study. To further enhance the translatability of the results, this investigation used a clinically relevant fractionated irradiation protocol (70 Gy over 35 fractions of 2 Gy each), which more closely mimics the standard protocol used in the head and neck cancer field. Furthermore, this study directly compared two contemporary restorative materials, which contain two different matrix architectures: S-PRG filler technology and organically modified siloxane network, which are fundamentally different, providing insight into the response of different restorative concepts to ionizing radiation. The use of validated surface measurement techniques, Vickers indentation for microhardness, and optical profilometry for surface roughness ensured the precision and reproducibility of measurements. Last, the controlled in vitro setting reduced the number of confounding variables and enabled a more accurate assessment of the radiation effects, apart from the intraoral variables.

From a clinical perspective, stable surface properties are important since alterations of the microhardness and surface roughness may have an impact on wear behavior, plaque deposition, and longevity of the restorations. The present results indicate that there is no significant effect of therapeutic radiation on surface roughness and microhardness of the Bioactive restorative material (Giomer) and organically modified ceramic (Ormocer) based composites. These findings suggest that both materials maintain stable surface characteristics when exposed to clinically relevant therapeutic radiation doses.

## Limitations

Certain limitations should be acknowledged. First, the study was conducted in vitro and therefore does not fully replicate the complex oral conditions of patients receiving head and neck radiotherapy. Second, only two restorative materials were evaluated, which may limit the generalizability of the findings. In addition, surface morphology was not assessed using advanced characterization techniques such as SEM or AFM, and surface evaluation was limited to the Ra parameter. Future studies incorporating artificial aging, thermocycling, mechanical loading, additional surface characterization methods, and clinical studies are recommended to validate these findings.

## Conclusion

Within the limitations of this in vitro study, therapeutic radiation at 70 Gy did not significantly compromise the surface microhardness or roughness of the tested Giomer-based (Beautifil II LS) and Ormocer-based (Admira Fusion) restorative materials. These findings suggest that both materials maintain stable surface characteristics when exposed to clinically relevant therapeutic radiation doses. However, broader investigation with additional material formulations and in vivo validation is necessary before generalizable clinical recommendations can be established. These findings contribute to the current evidence regarding the response of contemporary restorative materials to therapeutic ionizing radiation.

## Supplementary Information

Below is the link to the electronic supplementary material.

Supplementary Material 1

## Data Availability

The datasets used and/or analyzed during the current study are available from the corresponding author on reasonable request.
