# Supplementary material for: Effect of fractionated therapeutic radiotherapy on surface microhardness and roughness of giomer- and ormocer-based restorative composites: an in vitro study
Source: Sci Rep. 2026 Aug 5;16:24147. doi: 10.1038/s41598-026-64330-0 (PMC13443580; doi:10.1038/s41598-026-64330-0)
Supplement: Supplementary file 1 [file 41598_2026_64330_MOESM1_ESM.docx]

|  |  |
| --- | --- |

| **Supplementary Fig. S1** Specimens  stabilized in custom Styrofoam | **Supplementary Fig. S2**  Thermoplastic immobilization mask used in clinical radiotherapy | |
| --- | --- | --- |
|  |  | |
| **Supplementary Fig. S3** CT simulation | **Supplementary Fig. S4** Radiation treatment plan, showing dose distribution and target alignment | |
|  | |  |
|  | |  |

**Supplementary Fig. S5** Bar chart showing mean and standard deviation of microhardness for non-irradiated **(IR0)** and irradiated **(IR1)** for both restorative materials. not statistically significant (p > 0.05)

**Supplementary Fig. S6** Bar chart showing mean and standard deviation of surface roughness for non-irradiated (IR0) and irradiated (IR1) specimens of both restorative materials.
